# Emergence of related nontoxigenic Corynebacterium diphtheriae biotype mitis strains in Western Europe.

**DOI:** 10.3201/eid0503.990326

**Published:** 1999

**Authors:** G Funke, M Altwegg, L Frommelt, A von Graevenitz

**Affiliations:** University of Zurich, Switzerland.

## Abstract

We report on 17 isolates of Corynebacterium diphtheriae biotype mitis with related ribotypes from Switzerland, Germany, and France. Isolates came from skin and subcutaneous infections of injecting drug users, homeless persons, prisoners, and elderly orthopedic patients with joint prostheses or primary joint infections. Such isolates had only been observed in Switzerland.


### Emergence of Related Nontoxigenic *Corynebacterium diphtheriae* Biotype *mitis* Strains in Western Europe

**Guido Funke,* Martin Altwegg,* Lars Frommelt,† and Alexander von Graevenitz***
*University of Zurich, Zurich, Switzerland; and
†Endo-Klinik, Hamburg, Germany

We report on 17 isolates of *Corynebacterium diphtheriae* biotype *mitis* with related ribotypes from Switzerland, Germany, and France. Isolates came from skin and subcutaneous infections of injecting drug users, homeless persons, prisoners, and elderly orthopedic patients with joint prostheses or primary joint infections. Such isolates had only been observed in Switzerland.

Nontoxigenic *Corynebacterium diphtheriae* strains were recovered from approximately 1 per 1,000 throat swabs from immunized British military personnel in Germany from 1993 to 1995 (1). Such nontoxigenic *C. diphtheriae* biotype *mitis* isolates had been described in skin, throat, and blood cultures of Swiss injecting drug users; 32 of the isolates belonged to the same clone (2,3). Our study demonstrates that this clone and closely related clones occurred between 1990 and 1997 in two other European countries, and only sometimes in persons with poor hygiene.

Five *C. diphtheriae* isolates came from two laboratories in Zurich and Bern, Switzerland; 11 from four laboratories in Hamburg, Germany; and 1 from Paris, France (Table). They were identified in Zurich as *C. diphtheriae* biotype *mitis* (4); that is, they were nonlipophilic, were nitrate reductase-positive, and did not ferment glycogen. By polymerase chain reaction techniques (5), the diphtheria toxin gene was not detected in any isolate. For some isolates, the Elek test was also performed; it was consistently negative. For ribotyping, DNA was isolated, digested with either *Eco*RI or *Pvu*II, electrophoresed, blotted, and probed for rDNA as described elsewhere (2,3). Disk susceptibility testing to tetracycline and MIC determinations were done according to National Committee for Clinical Laboratory Standards methods (6). All other bacteria isolated were also identified and serotyped in Zurich.

Many (10 [59%] of 17) *C. diphtheriae* isolates were from skin or subcutaneous infections (wounds, ulcers) in Swiss patients and were found with *Staphylococcus aureus, Streptococcus pyogenes,* group C/G streptococci, or (sometimes) with gram-negative rods. Most patients in this subgroup were injecting drug users, homeless persons, prisoners, and (with one exception) men with a mean age of 40 (30 to 58) years. Another subgroup (from Germany) consisted of six patients with joint or bone infections (mentioned briefly in an earlier publication on coryneform bacteria from such infections; a seventh patient had *C. diphtheriae* biotype *gravis* [7]). The six had been hospitalized at the Endo Clinic in Hamburg, which specializes in orthopedic surgery. Four had had implantations of hip endoprostheses and had been admitted for prosthetic infections (with coagulase-negative staphylococci or *S. aureus)*; one pure culture of *C. diphtheriae* biotype *mitis* was obtained from a patient with a knee prosthesis, and one mixed culture with *Peptostreptococcus magnus* and *Peptostreptococcus prevotii* was obtained from a patient who had a purulent knee infection after a fracture. Their average age was 60 (23 to 82) years and, to our knowledge, none was a drug user. While the Hamburg isolates were recovered only once from every patient,

Address for correspondence: Alexander von Graevenitz, Department of Medical Microbiology, University of Zurich, Gloriastrasse 32, CH-8028 Zurich, Switzerland; fax: 411-634-4906; e-mail: avg@immv.unizh.ch.

Table. Nontoxigenic *Corynebacterium diphtheriae* isolates

| Isolate no. | Date isolated[a] | Place of isolation[b] | Ribotyping pattern[c] *Eco*RI | *Pvu*II | Patient's sex, age (yr) | Clinical diagnosis/ source | Other bacteria isolated | Underlying conditions |
|---|---|---|---|---|---|---|---|---|
| 2012 | 1990 | Paris | A | C | m, 6 | Endocarditis/blood culture | ---- | Skin lesions, scabies |
| 2410 | 2/1995 | Hamburg | A | B | m, 30 | Ulcus/lower leg | *Staphylococcus aureus, Streptococcus pyogenes* | Prisoner |
| 2689 | 3/1995 | Hamburg | A | A | f, 82 | Puncture/hip joint | *S. epidermidis, Corynebacterium pseudodiphtheriticum* | Total hip joint endoprosthesis |
| 1682 | 9/1995 | Hamburg | A | B | m, 45 | Wound swab | *S. aureus, S. pyogenes* | Homelessness |
| 1935 | 3/1996 | Hamburg | A | B | m, 58 | Wound swab | *S. aureus, S. pyogenes* | Alcoholism, homelessness |
| 1836 | 3/1996 | Hamburg | A | B | m, 45 | Abscess/lower leg | *S. aureus, S. pyogenes* | Injecting drug use |
| 2661 | 3/1996 | Hamburg | A | A | m, 35 | Arthroscopy aspirate/knee joint | *Peptostreptococcus prevotii, P. magnus* | Osteosynthesis, arthrotomy, knee joint after trauma |
| 2658 | 4/1996 | Hamburg | A | A | f, 76 | Fistula/hip joint | *S. aureus* | Total hip joint endoprosthesis |
| 2674 | 5/1996 | Hamburg | A | A | f, 62 | Aspirate/hip joint | *Streptococcus* group C | Total hip joint endoprosthesis |
| 2670 | 7/1996 | Hamburg | A | A | f, 81 | Aspirate/knee joint | ---- | Total knee joint endoprosthesis |
| 2667 | 7/1996 | Hamburg | A | A | m, 23 | Swab/hip joint | Coagulase-negative staphylococci | Total hip joint endoprosthesis |
| 2413 | 11/1996 | Hamburg | A | B | m, 36 | Ulcus/lower leg | *S. aureus, Streptococcus* group C/G | Prisoner |
| 2446 | 12/1996 | Zurich | A | A | f, 38 | Wound/upper leg | *S. aureus, S. pyogenes* | Injecting drug use |
| 2464 | 1/1997 | Zurich | A | A | m, 39 | Ulcera/lower arm | *Escherichia coli, S. aureus, S. pyogenes* | Injecting drug use |
| 2473 | 1/1997 | Zurich | A | A | m, 38 | Ulcera/leg | *E. coli, Streptococcus* group C, G | Injecting drug use |
| 2475 | 1/1997 | Zurich | A | A | m, 31 | Ulcus/pretibial | *Pseudomonas aeruginosa, E. coli, Streptococcus* group C, G | Injecting drug use |
| 480 | 5/1997 | Bern | A | A | m, 39 | Ulcus/upper leg | *S. aureus, Clostridium perfringens,* mixed anaerobic flora | Injecting drug use |

[a]Month/Year
[b]Paris, France; Hamburg, Germany; Zurich, Switzerland; Bern, Switzerland.
[c]DNA was isolated, digested with either *Eco*RI or *Pvu*II, electrophoresed, blotted, and probed for DNA. Three distinct ribopatterns emerged, each with eight bands: patterns A, B, and C.

their isolation dates stretched from March 1, 1995, until July 26, 1996. Three were isolated on the day of admission, and all of them were isolated by different technologists. Finally, one child (from France) had endocarditis with *C. diphtheriae* biotype *mitis*.

All isolates had identical antimicrobial susceptibility patterns. They were susceptible to amoxicillin (MIC, 0.25 µg/ml), amoxicillin-clavulanic acid (0.06 µg/ml), chloramphenicol (2 µg/ml), ciprofloxacin (0.25 µg/ml), clarithromycin (0.03 µg/ml), clindamycin (0.25 µg/ml), imipenem (0.03 µg/ml), penicillin (0.25 µg/ml), and vancomycin (1 µg/ml). In contrast, all strains were resistant to tetracycline in the disk diffusion test (inhibition zone diameter 10 mm to 11 mm); their MICs for tetracycline, doxycycline, and minocycline were 64 µg/ml, 16 µg/ml, and 16 µg/ml, respectively. While this type of isolated tetracycline resistance was typical for the isolates from Swiss injecting drug users (3), tetracycline resistance in nontoxigenic *C. diphtheriae* has otherwise very rarely been observed in Europe (8). It has, however, been reported in toxigenic *C. diphtheriae* isolates from Indonesia and, rarely, from Canada (9). The mechanism conferring this resistance in our strains has not been investigated.

On analysis with restriction enzyme *Pvu* II, three different ribopatterns with eight bands each were found among the strains (Figure). These patterns, though distinct, shared five (A vs. C, B vs. C) and six (B vs. A) bands, respectively, and, therefore, may be considered related, as they would be if criteria for pulsed-field gel electrophoresis were applied (10). This view is supported by the fact that all strains had identical *Eco*RI patterns (not shown). All Swiss isolates were identical with the use of both enzymes, whereas both the Swiss pattern and a second pattern were found among the Hamburg isolates. The third pattern was found exclusively in the single French strain. The staphylococcal and streptococcal strains were not typed; they are no longer available to us.

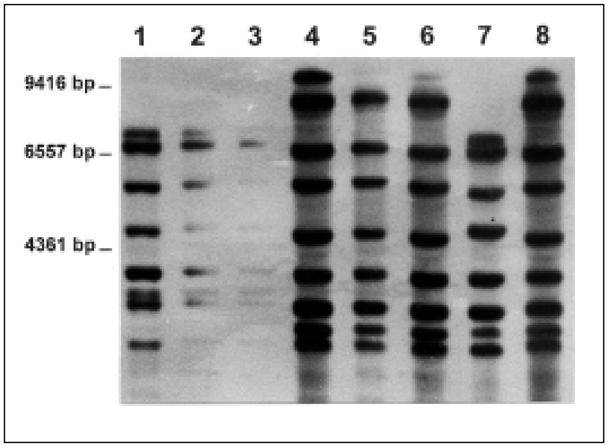

Figure. Ribosomal RNA gene restriction patterns obtained by using restriction enzyme Pvu II of *Corynebacterium diphtheriae* isolates belonging to ribotypes B (lanes 1 to 3), A (lanes 4 to 6 and 8), and C (lane 7).

Our isolates thus resemble those reported from Switzerland between 1990 and early 1996 (2,3), which were also often accompanied by *S. aureus* or beta-hemolytic streptococci. Such nontoxigenic *C. diphtheriae mitis* may cause endocarditis, arthritis, and osteomyelitis (11,12). Most of the 52 isolates from France (11), examined with restriction enzymes different from ours and not available to us, also belonged to one ribotype. Their relatedness to our strains is unknown; however, they were largely tetracycline-susceptible. The two throat isolates from St. Petersburg, Russia, associated with a fatal diphtherialike disease (12) were not typed or tested for antibiotic susceptibility.

The origin of the isolates we describe is unknown. They may have been present (but unrecognized) in the population for a long time. The mode of transmission is most likely common use of drug paraphernalia in the injecting drug use cases and in the endocarditis case; transmission is very difficult to explain in the orthopedic infection subgroup. Although the Swiss and the Danish borders are not close, migration and contacts are not uncommon among injecting drug users. The isolates may also have been distributed through the drugs themselves, as recently reported for *S. pyogenes* in Switzerland (13).

Our study may be representative for Switzerland and Germany, but since submitting *C. diphtheriae* strains to a central reference laboratory in these countries is not mandatory, we cannot estimate the frequency of these strains. These strains may also have spread to other European countries; this hypothesis can only be tested in a large multicenter study of European diphtheria reference laboratories.

### Acknowledgments

We thank J. Lüthy-Hottenstein and V. Pünter-Streit for excellent technical assistance and P. Das for her help in securing the strains from Hamburg. G. Funke is recipient of a European Society for Clinical Microbiology and Infectious Diseases research fellowship.

Dr. Funke is director of clinical microbiology at Gärtner & Colleagues Laboratories in Weingarten, Germany. His areas of expertise are clinical and systematic bacteriology. Research interests include taxonomy and disease associations of coryneform bacteria and actinomycetes, as well as rapid methods for identification and susceptibility testing of medically relevant bacteria.

### References

1. Sloss JM, Hunjan RS. Incidence of non-toxigenic corynebacteria diphtheria in British military personnel in Germany. J Infect 1996;33:139.
2. Gubler J, Huber-Schneider C, Gruner E, Altwegg M. An outbreak of non-toxigenic *Corynebacterium diphtheriae* infection: single bacterial clone causing invasive infection among Swiss drug users. Clin Infect Dis 1998;27:1295-8.
3. Gruner E, Zuber PLF, Martinetti-Lucchini G, von Graevenitz A, Altwegg M. A cluster of non-toxigenic *Corynebacterium diphtheriae* infections among Swiss intravenous drug abusers. Medical Microbiology Letters 1992;1:160-7.
4. Von Graevenitz A, Funke G. An identification scheme for rapidly and aerobically growing Gram-positive rods. Zentralbl Bakteriol 1996;284:246-54.

5. Martinetti-Lucchini G, Gruner E, Altwegg M. Rapid detection of diphtheria toxin by the polymerase chain reaction. Medical Microbiology Letters 1992;1:276-83.
6. National Committee for Clinical Laboratory Standards. Performance standards for antimicrobial susceptibility testing; eighth informational supplement [NCCLS document M 100-S8]. Wayne (PA): The Committee; 1998.
7. von Graevenitz A, Frommelt L, Pünter-Streit V, Funke G. Diversity of coryneforms found in infections following prosthetic joint insertion and open fractures. Infection 1998;26:36-8.
8. Patey O, Bimet F, Emond JP, Estrangin E, Riegel P, Halioua B, et al. Antibiotic susceptibilities of 38 non-toxigenic strains of *Corynebacterium diphtheriae.* J Antimicrob Chemother 1995;36:1108-10.
9. Rockhill RC, Sumarmo, Hadiputranto H, Siregar SP, Muslihun B. Tetracycline resistance of *Corynebacterium diphtheriae* isolated from diphtheria patients in Jakarta, Indonesia. Antimicrob Agents Chemother 1982;21:842-3.
10. Tenover FC, Arbeit RD, Goering RV, Mickelsen PA, Murray BE, Persing DH, et al. Interpreting chromosomal DNA restriction patterns produced by pulsed-field gel electrophoresis: criteria for bacterial strain typing. J Clin Microbiol 1995;33:2233-9.
11. Patey O, Bimet F, Riegel P, Halioua B, Emond JP, Estrangin E, et al. Clinical and molecular study of *Corynebacterium diphtheriae* systemic infections in France. J Clin Microbiol 1997;35:441-5.
12. Rakhmanova AG, Lumio J, Groundstroem KWE, Taits BM, Zinserling VA, Kadyrova SN, et al. Fatal respiratory tract diphtheria apparently caused by nontoxigenic strains of *Corynebacterium diphtheriae.* Eur J Clin Microbiol Infect Dis 1997;16:816-20.
13. Streptokokken-Infektionen bei Drogensüchtigen in der Region Bern. Bulletin des Bundesamtes für Gesundheit 1997;44:3.

